# Do outcomes differ between work and non-work-related injury in a universal injury compensation system? Findings from the New Zealand Prospective Outcomes of Injury Study

**DOI:** 10.1186/1471-2458-13-995

**Published:** 2013-10-22

**Authors:** Rebbecca Lilley, Gabrielle Davie, John Langley, Shanthi Ameratunga, Sarah Derrett

**Affiliations:** 1 Injury Prevention Research Unit, Department of Preventive and Social Medicine, Dunedin School of Medicine, University of Otago, Dunedin, New Zealand; 2 Section of Epidemiology and Biostatistics, School of Population Health, Faculty of Medical and Health Science, University of Auckland, Auckland, New Zealand; 3 School of Health and Social Services, College of Health, Massey University, Palmerston North, New Zealand

**Keywords:** Work-related injury, Non-work injury, Recovery, Rehabilitation, Injury, Compensation, Outcome, Prospective cohort

## Abstract

**Background:**

Poorer recovery outcomes for workers injured in a work setting, as opposed to a non-work setting, are commonly attributed to differences in financial gain via entitlement to compensation by injury setting (ie. workers compensation schemes). To date, this attribution hasn’t been tested in a situation where both work and non-work-related injuries have an equivalent entitlement to compensation. This study tests the hypothesis that there will be no differences in recovery outcomes for workers by injury setting (work and non-work) within a single universal entitlement injury compensation scheme.

**Methods:**

Workforce active participants from the Prospective Outcomes of Injury Study (POIS) cohort were followed up at 3- and 12-months following injury. Participants who were injured in the period June 2007- May 2009 were recruited from New Zealand’s universal entitlement injury compensation scheme managed by the Accident Compensation Corporation (ACC). An analysis of ten vocational, disability, functional and psychological recovery outcomes was undertaken by injury setting. Modified Poisson regression analyses were undertaken to examine the relationship between injury setting and recovery outcomes.

**Results:**

Of 2092 eligible participants, 741 (35%) had sustained an injury in a work setting. At 3 months, workers with work-related injuries had an elevated risk of work absence however, this difference disappeared after controlling for confounding variables (adjusted RR 1.10, 95% CI 0.94-1.29). By 12 months, workers with work-related injuries had poorer recovery outcomes with a higher risk of absence from work (aRR 1.37, 95% CI 1.10-1.70), mobility-related functional problems (aRR 1.35, 95% CI 1.14-1.60), disability (aRR 1.32, 95% CI 1.04-1.68) and impaired functioning related to anxiety/depression (aRR 1.21, 95% CI 1.00-1.46).

**Conclusion:**

Our study, comparing recovery outcomes for workers by injury setting within a single universal entitlement injury compensation scheme, found mixed support for the hypothesis tested. After adjustment for possible covariates recovery outcomes did not differ by injury setting at 3 months following injury, however, by 12 months vocational, disability and some functional outcomes, were poorer for workers with work-related injuries. Given our findings, and other potential mechanisms for poorer outcomes for workers with work-related injuries, further research beyond differences in entitlement to compensation should be undertaken to inform future interventions.

## Background

It is commonly argued that workers recover from work-related injury more slowly than those with similar injuries sustained in a non-work injury setting. Previous comparisons of outcomes following work-related and non-work-related injuries have found differences in outcomes by injury setting with those sustaining work-related injuries more likely to have poorer vocational, psychological, neurological, functional, psychosocial and general health outcomes [1-4]. It is difficult to generalise from these findings as these studies had different inclusion criteria, measurement instruments, types and severities of injury examined, timing of follow-up points, and limited opportunity to examine a broad range of covariates. Differences in recovery outcomes for workers injured in work settings are often attributed to secondary financial gain via workers compensation insurance or litigation, as the non-work comparison group is usually generated from a population with vastly different entitlements to compensation [2,3]. Entitlement to compensation is known to be associated with slower recovery from injury [5,6]. For example, processes for establishing entitlement in some schemes can lead to poorer outcomes for those with compensable injuries due to difficulties in accessing financial compensation, treatment, and the resulting psychological distress [7].

Previous analyses of differences in outcomes by injury setting have been limited by the absence of a comparably compensated non-work-related injury group. New Zealand’s universal Injury Compensation and Rehabilitation scheme, managed by the Accident Compensation Corporation (ACC), is unique as it provides compensation and rehabilitation services regardless of the cause or setting of injury (ie. injury sustained at home, recreationally, on road, or at work) with all workers eligible for earnings-related compensation up to 80% of their weekly earnings after a week’s absence from work, as well as treatment, vocational and rehabilitation services. The Prospective Outcomes of Injury Study (POIS) recruited participants from the ACC providing the opportunity to examine potential differences in outcomes for those with injuries sustained in a work or non-work setting within a single compensation scheme. This paper examines whether or not differences in vocational, disability, functional and psychological recovery outcomes exist 3- and 12-months after injury between participants who sustained an injury while engaged in work and participants injured in other non-work settings when entitlement to earnings-related compensation is equivalent.

The hypothesis to be tested in this paper is that there will be no difference in recovery outcomes for workers by injury setting (work-related and non-work related) within New Zealand’s universal entitlement injury entitlement scheme.

## Methods

### Study sample & data collection

The POIS cohort of 2856 participants, followed up at 3-, 5-, 12- and 24-months after injury, was recruited from New Zealand’s universal, no-fault ACC scheme [8]. This paper examines outcomes collected at 3- and 12-months following injury for the 2626 (92%) participants who indicated they were actively working in paid employment such as for salary, wages or self-employed earnings (referred to as workforce active) prior to their injury. Data collected at 3 months was of interest to examine short-term outcomes, while 12 month data was included to examine if there were differences in medium-term outcomes. Outcome data to 24 months was unavailable at the time of analysis. Injuries were sustained by participants in various recreational, road, public, home and workplace settings. Those eligible for inclusion were people aged 18 to 64 years of age, injured in the period June 2007 to May 2009 who consulted with a primary or secondary healthcare professional and were subsequently registered on the ACC entitlement claimant register indicating the likelihood of requiring more than simple medical treatment. Ethical approval was granted from New Zealand’s Health and Disability Multi-Regional Ethics Committee (MEC/07/07/093). Consent was obtained verbally from each participant. More detail on the recruitment protocol is provided elsewhere [9].

### Explanatory variables

The main explanatory variable of interest was whether or not the injury event was work-related. Work-related injury was defined as injury sustained while engaged in a work activity for financial gain, or while commuting to or from work. Non-work-related injury is all other injury, including injuries sustained on the road, at home or in recreational settings.

Other explanatory variables were considered as possible a-priori confounders. Pre-injury socio-demographic characteristics included age at time of first interview, gender, highest educational qualification, personal income, and occupation based on questions from the New Zealand Census [10]. Education was grouped as “no formal”, “secondary” and “post-secondary” educational qualifications. Personal income was calculated as an annual gross amount (New Zealand dollars) and grouped into “no income given”, “≤ $30,000”, “$30,001-$50,000”, and “≥$50,001”. Occupation was classified using the New Zealand Standard Classification of Occupation (NZSCO) [11] and then further grouped into: professional (NZSCO Major level 1–3), technical (NZSCO Major level 4–5), trade and manual (NZSCO Major level 6–9) and unclassified occupations. Employment status was based upon a modified single question from the European Survey on Working Conditions “In your main job before your injury were you: a paid employee; self-employed and not employing others; or an employer of other person(s) in your own business?”, grouped into: employee, self-employed and employer [12].

Pre-injury health characteristics included co-morbidities assessed using a modified instrument from the New Zealand Health Survey [13]. Responses were grouped based upon the number of co-morbidities reported by participants from the 22 listed diagnosed conditions: none; 1; and 2 or more co-morbidities.

All available injury diagnoses for each participant were obtained from ACC [14]. Rather than restrict injuries to a primary diagnosis, we used all available injury diagnoses for an individual. Based on the ICD-10 injury mortality diagnosis matrix and the Barell injury diagnosis matrix, [15,16] eleven binary variables indicating whether the participant sustained an injury of particular type and region were identified: head, neck and intracranial injury; head & neck superficial injury; upper extremity fracture; upper extremity open wound; upper extremity superficial injury; upper body strain or sprain; spine dislocation, sprain or strain; lower extremity fracture; lower extremity open wound; lower extremity superficial injury; and lower body strain or sprain based upon prevalence of combination of nature and body region [14]. For example, for the upper extremity fracture variable the “yes” category contains anyone who sustained an upper extremity fracture. This will include participants for whom this was their only diagnosis in additional to participants who had other injury diagnoses, such as lower extremity fracture or internal organ damage. The New Injury Severity Score (NISS), an injury severity measure based on anatomical damage, was created by first mapping ICD-10 injury codes to the Abbreviated Injury Scale (AIS), with the three highest AIS scores transformed into NISS by summing the squares of these values [17,18]. For this paper, anatomical severity-NISS is grouped into 3 categories: NISS 1–3 (AIS-1 injuries only), NISS 4–6 (one AIS 2 injury and possibly additional AIS-1 injuries) and NISS >6 (at least two AIS-2 injuries or one AIS-3 or greater injury). Hospital admission within 7 days of the injury event, was assessed using probabilistic linkage to New Zealand’s National Minimum Data Set (NMDS), which captures discharge data on emergency department treatment for 3 hours or more, or hospital admission. Perceived threat to life and perceived threat of severe disability were assessed from interview data using the single items: “At the time, did you feel your injury was a threat to your life?” and “At the time, did you feel the injury was a threat of severe long-term disability to you?” Responses for both questions have been dichotomised into: yes/maybe, no. The question “Did you have difficulties getting to or accessing healthcare services” was used to create the variable 'access to healthcare services’ by dichotomising responses into: trouble accessing (yes-trouble and mixed) and no trouble accessing (no-trouble). Earnings-related compensation payment was assessed using ACC data for each participant: those receiving any amount of compensation for lost earnings from ACC were considered to have received earnings-related compensation.

Pre-injury measures of the WHODAS disability [19], and the five EQ-5D [20] and additional cognitive functional [21] outcome measures were available to examine possible differences in pre-injury disability and functional status by injury setting. At the 3 month interview participants were asked to recall their pre-injury disability and functional status for the 30 days prior to their injury for disability and in the day prior to their injury for functional status. For further description of these variables see the outcome variables section below.

### Outcome variables

Ten outcomes were assessed at both 3- and 12-month post injury interviews including one each of vocational and disability outcomes, six functional outcomes, and two psychological outcomes.

Vocational outcome was assessed using work status ascertained at the 3 month interview with the single item “Are you back at work following your injury?” with responses yes and no. At the 12 month interview work status was ascertained using the single item “which of the following best describes your paid work situation now?” with participants indicating full-time and part-time work for pay considered to be working, and those responding receiving a benefit and/or ACC compensation or indicating unemployment considered to be absent from work.

The disability outcome was assessed at both 3- and 12-months using the WHODAS simple summed score (total score range 0–48) calculated using the brief WHODAS II 12-item instrument [19,22]. Participants were asked to report any difficulties with 12 activity and participation items in the 30 days prior to interview. The WHODAS simple summed score was dichotomised into disability (≥10) and no (or lesser) disability (<10) [22], as described previously [14].

Functional outcomes were assessed using the EQ-5D health state classification system at both 3- and 12-months [20]. The five EQ-5D dimensions (mobility, self-care, usual activities, pain/discomfort and anxiety/depression), plus an additional cognitive dimension [21], were examined as individual outcomes. Responses were dichotomised into problematic (some or extreme problems) and non-problematic (no problems).

Two measures of psychological outcome were used. Kessler-6 psychological distress for the 30 days prior to interview was measured at both 3- and 12-months using the Kessler-6 summary score with scores dichotomised into distressed (≥13) and not distressed (<13) [23]. Post-traumatic Stress Disorder (PTSD) was assessed at the 12 month interview only using the Impact of Event Scale total score, using ≥27 as the threshold for the indication of PTSD [24].

### Statistical analysis

Analyses compared the two injury groups (work-related and non-work-related) for differences in outcomes using chi-square tests. Modified Poisson regression models using robust error variance were used to estimate relative risks using the ten dichotomised outcomes and work-related injury as the main explanatory variable [25]. Pre-injury socio-demographic, co-morbidities, and injury (including anatomical severity-NISS and hospital admission) covariates, selected on the basis of an a priori relationship with work-related injury and/or identification in previous POIS analyses, were examined for inclusion into multivariable models by examining differences in distribution of the covariates by injury setting. Covariates with a p-value <0.1 were included across all the multivariable models. Pre-injury disability and functional outcome measures were adjusted for in the corresponding disability and functional outcome models. Separate unadjusted and adjusted models were created for each outcome, for both the 3- and 12-month periods. Analysis was undertaken using STATA v11.1 [26].

## Results

Of the 2626 workforce active participants, 2089 participants had completed both 3- and 12-month interviews. 741 (35%) of injuries were sustained in a work-related setting.

Univariate analyses of differences in socio-demographic and pre-existing co-morbidities, according to whether or not the injury was work-related, are presented in Table 1. Participants with work-related injuries were more likely to be aged over 45 years, male, have post-secondary educational qualifications, were less likely to be in the highest income group, more likely to be self-employed or an employer, less likely to be in professional or technical occupations and more likely to work in a manual occupation compared to those with non-work-related injuries.

**Table 1:** **Socio**-**demographic and pre**-**existing co**-**morbidity characteristics according to injury setting** (**N**=**2089**)

**Characteristic**	**Work (n=741) n (%)**	**Non-work (n=1348) n (%)**	**p-value**
Age			
18–24	55 (7)	160 (12)	<0.001
25–34	109 (15)	305 (23)	
35–44	170 (23)	309 (23)	
45–54	232 (31)	339 (25)	
55-64	175 (24)	235 (17)	
Gender			
Male	517 (70)	768 (57)	<0.001
Female	224 (30)	580 (43)	
Highest Educational Qualification*			
Post-secondary qualifications	133 (18)	172 (13)	0.004
Secondary qualifications	167 (23)	323 (24)	
No formal qualifications	426 (59)	837 (63)	
Personal Income (NZ$)			
≥$50,001	207 (28)	469 (35)	0.01
$30,001-$50,000	261 (35)	421 (31)	
≤$30,000	159 (21)	257 (19)	
No income given	114 (15)	201 (15)	
Occupation*			
Professional	182 (25)	589 (43)	<0.001
Technical	113 (15)	335 (25)	
Trade/manual	415 (56)	382 (28)	
Unclassified	26 (4)	38 (3)	
Employment Status*			
Employee	600 (81)	1156 (86)	0.02
Self-employed	91 (12)	11 (10)	
Employer	48 (7)	61 (4)	
Prior co-morbidities*			
None	336 (51)	705 (54)	0.2
1	196 (27)	354 (27)	
2 or more	161 (22)	252 (19)	

*Numbers do not sum to N=2089 for some variables due to missing values.

Differences in injury and compensation related variables by injury setting are presented in Table 2. There were a higher proportion of dislocations, sprains and strains of the spine and upper extremity open wounds in those with work-related injury. Conversely the prevalence of upper and lower extremity fractures, hospital admission and injuries with an anatomical severity of NISS 4–6 was greater in those with non-work-related injury. Receipt of earnings-related compensation was greater in those with work-related injury.

**Table 2:** **Injury characteristics according to injury setting** (**N**=**2089**)

**Injury characteristic**	**Work (n=741) n (%)**	**Non-work (n=1348) n (%)**	**p-value**
Lower extremity fracture			
No	653 (88)	1073 (80)	<0.001
Yes	88 (12)	275 (20)	
Upper extremity fracture			
No	644 (86)	1078 (80)	<0.001
Yes	97 (13)	270 (20)	
Upper body strain, sprain & dislocation			
No	625 (84)	1175 (87)	0.07
Yes	116 (16)	173 (13)	
Lower body strain, sprain & dislocation			
No	605 (82)	966 (72)	<0.001
Yes	136 (18)	382 (28)	
Spine strain, sprain & dislocation			
No	563 (76)	1182 (88)	<0.001
Yes	178 (24)	166 (12)	
Head, neck & intracranial			
No	718 (94)	1297 (96)	0.4
Yes	23 (3)	51 (4)	
Lower extremity open wound			
No	707 (95)	1297 (96)	0.3
Yes	34 (5)	51 (4)	
Upper extremity open wound			
No	684 (92)	1276 (95)	0.03
Yes	57 (8)	27 (5)	
Lower extremity superficial injury			
No	687 (93)	1260 (93)	0.5
Yes	54 (7)	88 (7)	
Upper extremity superficial injury			
No	701 (95)	1286 (95)	0.4
Yes	40 (5)	62 (5)	
Head or neck superficial injury			
No	713 (96)	1304 (97)	0.5
Yes	28 (4)	44 (3)	
Hospital admission			
No	576 (78)	997 (74)	0.05
Yes	165 (22)	351 (26)	
Anatomical severity-NISS			
1–3	368 (52)	461 (35)	<0.001
4–6	244 (34)	651 (50)	
>6	97 (14)	203 (15)	
Perceived threat to life			
No threat	646 (89)	1187 (90)	0.5
Yes/maybe a threat	83 (11)	139 (10)	
Perceived threat severe disability			
No threat	420 (58)	814 (62)	0.08
Yes/maybe a threat	309 (42)	509 (38)	
Access to healthcare services			
No trouble accessing	669 (91)	1203 (90)	0.3
Trouble accessing	63 (9)	132 (10)	
Earnings-related compensation received			
No	72 (10)	311 (23)	<0.001
Yes	669 (90)	1037 (76)	

Missing values are not individually stated.

Table 3 presents the univariate analyses for all ten outcomes examined. Participants who had work-related injuries reported higher prevalence of work absence 3 months following injury. Differences in other outcomes at 3 months between the work and non-work groups were not statistically significant. Despite improvement in most outcomes by 12 months after injury, different patterns of recovery are observed between the work-related and non-work-related injury groups. Participants with work-related injuries reported at 12 months higher prevalence of poor vocational, disability, functional and PTSD health outcomes, and a lower prevalence of Kessler-6 psychological distress compared with workers with non-work injuries.

**Table 3:** **Comparisons of outcomes according to injury setting at 3**- **and 12**-**months following injury**

**Outcomes**	**Work n (%)**	**Non-work n (%)**	**p value**
**3 month outcomes**			
**Vocational**			
Absent from work (Y)	232 (31)	316 (24)	<0.001
**Disability**			
WHO DAS (≥10)	305 (42)	541 (41)	0.7
**Functional**			
Problems with mobility (EQ1)	308 (42)	558 (41)	0.9
Problems with self-care (EQ2)	174 (23)	322 (24)	0.8
Problems with usual activities (EQ3)	375 (51)	732 (54)	0.1
Pain/Discomfort (EQ4)	497 (67)	934 (67)	0.2
Anxiety/Depression (EQ5)	164 (22)	274 (20)	0.3
Problems with cognitive ability	110 (13)	178 (15)	0.3
**Psychological**			
Psychological distress (≥13)	57 (8)	85 (6)	0.2
**12 month outcomes**			
**Vocational**			
Absent from work (Y)	155 (21)	174 (13)	<0.001
**Disability**			
WHO DAS (≥10)	132 (18)	150 (11)	<0.001
**Functional**			
Problems with mobility (EQ1)	206 (28)	268 (20)	<0.001
Problems with self-care (EQ2)	71 (10)	73 (5)	<0.001
Problems with usual activities (EQ3)	243 (33)	359 (27)	0.002
Pain/Discomfort (EQ4)	405 (55)	660 (49)	0.01
Anxiety/Depression (EQ5)	170 (23)	240 (18)	0.004
Problems with cognitive ability	132 (18)	185 (14)	0.01
**Psychological**			
Psychological distress (≥13)	39 (5)	45 (3)	0.03
PTSD (≥27)	116 (16)	169 (13)	0.05

Table 4 presents the multivariable analyses for all ten health outcomes. At 3 months following injury only work absence was associated with work-related injury (RR 1.33, 95% CI 1.15-1.54), however, after controlling for the socio-demographic, pre-existing health, injury and compensation differences between the two groups this association was attenuated (aRR 1.10, 95% CI 0.94-1.29). Those with a work-related injury showed a tendency towards a reduced risk of problems due to usual activities and pain/discomfort but this was not statistically significant with either the unadjusted or adjusted relative risks.

**Table 4:** **Multivariable associations between injury setting and recovery outcomes at 3**- **and 12**-**months following injury**

**Outcomes**	**Non-work**	**Work RR (95% CI)**	**Non-work**	Work aRR* (95% CI)
**3 month outcomes**				
**Vocational**				
Absent from work (Y)	Ref	**1.33 (1.15-1.54)**	Ref	1.10 (0.94-1.29)
**Disability**				
WHO DAS (≥10)	Ref	1.02 (0.92-1.14)	Ref	0.99 (0.88-1.11)
**Functional**				
Problems with mobility (EQ1)	Ref	1.00 (0.90-1.11)	Ref	1.03 (0.92-1.14)
Problems with self-care (EQ2)	Ref	0.98 (0.84-1.15)	Ref	0.90 (0.75-1.07)
Problems with usual activities (EQ3)	Ref	0.93 (0.85-1.02)	Ref	0.94 (0.86-1.03)
Pain/Discomfort (EQ4)	Ref	0.96 (0.91-1.03)	Ref	0.96 (0.90-1.03)
Anxiety/Depression (EQ5)	Ref	1.09 (0.92-1.29)	Ref	1.00 (0.82-1.20)
Problems with cognitive ability	Ref	1.12 (0.90-1.40)	Ref	0.94 (0.74-1.21)
**Psychological**				
Psychological distress (≥13)	Ref	1.22 (0.88-1.69)	Ref	1.23 (0.85-1.78)
**12 month outcomes**				
**Vocational**				
Absent from work (Y)	Ref	**1.61 (1.33-1.98)**	Ref	**1.37 (1.10-1.70)**
**Disability**				
WHO DAS (≥10)	Ref	**1.62 (1.30-2.01)**	Ref	**1.32 (1.04-1.68)**
**Functional**				
Problems with mobility (EQ1)	Ref	**1.40 (1.20-1.64)**	Ref	**1.35 (1.14-1.60)**
Problems with self-care (EQ2)	Ref	**1.78 (1.30-2.44)**	Ref	1.38 (0.98-1.95)
Problems with usual activities (EQ3)	Ref	**1.23 (1.08-1.42)**	Ref	1.14 (0.98-1.31)
Pain/Discomfort (EQ4)	Ref	**1.11 (1.03-1.22)**	Ref	1.06 (0.97-1.17)
Anxiety/Depression (EQ5)	Ref	**1.29 (1.09-1.54)**	Ref	**1.21 (1.00-1.46)**
Problems with cognitive ability	Ref	**1.30 (1.05-1.60)**	Ref	1.18 (0.94-1.48)
**Psychological**				
Psychological distress (≥13)	Ref	**1.58 (1.04-2.40)**	Ref	1.44 (0.92-2.25)
PTSD (≥27)	Ref	**1.24 (1.00-1.55)**	Ref	1.06 (0.83-1.35)

Key: *RR*=Relative risk aRR*= Adjusted relative risk: Adjusted for age; gender; occupation; income; highest educational qualification; employment status; lower extremity fracture; upper extremity fracture; upper body strain or sprain; spinal dislocation, sprain or strain; hospital admission; anatomical severity-NISS; threat of serious disability; and receipt of earnings-related compensation. The disability and six functional outcome models were additionally adjusted for pre-injury measures of the outcome.

At 12 months all outcomes examined, had a significantly higher unadjusted risk of poor recovery outcomes for those injured in a work-setting (Table 4). After controlling for socio-demographic, pre-existing health, injury and compensation differences between the groups, significantly higher risk of work absence (aRR 1.37, 95% CI 1.10-1.70), disability (aRR 1.32, 95% CI 1.04-1.68), and problems related to mobility (aRR 1.35, 95% CI 1.14-1.60) were found for those workers with work-related injuries. Workers with work-related injury were also at higher risk of anxiety/depression (aRR 1.21, 95% CI 1.00-1.46), but this relationship was weaker as the 95% confidence interval just captures the null value of 1. Workers with work-related injuries also showed a tendency towards increased risk of functional problems related to usual activity (aRR 1.14, 95% CI 0.98-1.31), functional problems related to self-care (aRR 1.38, 95% CI 0.98-1.95), and functional problems due to pain/discomfort (aRR 1.06, 95% CI 0.97-1.17), however, for these, the 95% confidence intervals capture the null value. After adjusting for covariates, the trends in the same direction for psychological outcomes were statistically non-significant.

## Discussion

The results of this study indicate mixed support for our hypothesis that there will be no differences in recovery outcomes by injury setting within New Zealand’s universal injury compensation context. A time-dependent pattern of recovery was observed in our study. At 3 months following injury our study found just one outcome was associated with injury setting; those injured in a work setting being at 33% increased unadjusted risk of being absent from work compared to those with non-work-place injuries. However, no differences in the risk were observed once socio-demographic, pre-existing health or disability conditions, injury and compensation differences were controlled for in multi-variable analyses, thus providing no evidence to reject our hypothesis of there being no difference in recovery outcome by injury setting within the New Zealand universal compensation context at this time point.

At 12 months following injury the prevalence of recovery problems decreased for all outcomes examined, with the exception of functional difficulties due to anxiety/depression (which remained constant) and cognitive problems where the prevalence increased for those with work-related injuries. By 12 months a different pattern of recovery is apparent according to injury setting. After adjustment for covariates, multivariable analyses indicated workers with work-related injuries compared to those injured in a non-work setting, were at increased risk of poor outcomes although this relationship was statistically significant for only three of the ten outcomes. Particularly strong relationships were found for work absence and mobility problems with workers injured in a work-related setting estimated to be at 37% and 35% respectively greater risk of poor outcomes. This suggests that mechanisms for poorer outcomes at 12 months may exist rather than differences in financial gain due to compensation entitlement.

Comparisons of our findings with previous studies examining differences in outcomes by injury setting are limited by a number of methodological issues outlined in the introduction. Acknowledging this, our findings are broadly consistent with previous studies which have demonstrated that while most outcomes improve with increasing time of follow-up, patterns of recovery were slower for those with work-related injuries [1-4,27]. At 12 months our study observed poorer outcomes for workers, which does not support the commonly-raised explanation of financial gain (consequent to different compensation entitlements between workers and non-workers) as the reason for poorer outcomes among those injured in a work setting.

Given that both injury settings compared in our study have the same entitlement to compensation and rehabilitation from New Zealand’s universal accident compensation scheme, other possible mechanisms for poorer outcomes in workers with work-related injuries need to be considered. For example, MacEachen et al., identified five potential explanations for delayed return to work: 1) patterns of pain and coping; 2) interface with the healthcare system; 3) workplace relationships; 4) the compensation system; and 5) workers experiences, that are worthy of further examination as they may differ by injury setting [28].

The relationship between the worker, workplace and the functioning of workplace rehabilitation systems has been identified as a barrier to recovery with employer-related factors, such as the reluctance or an inability to offer workplace accommodations, shown to impede work participation [29]. It is possible that an employer’s ability or willingness to accommodate injured workers is less in situations where workers sustained their injury while at the workplace or while the worker still carries some functional or disability limitations. Likewise, a worker injured at the workplace may also be more reluctant to be re-exposed to those workplace hazards that caused the original injury, especially if those hazards have not been modified since the injury event, or while functional or disability limitations are present. A study of hand injuries observed PTSD was most likely to impact upon those who sustained their hand injury at work [30]. While there was no evidence of a significant difference in PTSD outcomes after substantial adjustment for covariates between the two groups, it is possible that return to the site of injury is an explanation for our observed differences in work absence 12 months after injury. Workers injured at work may also be subject to elevated levels of suspicion by compensation case managers, health professionals, employers, co-workers and other members of society delaying recovery [28]. For example, some employers may consider a workplace injury as a problematic worker issue [28]. Future research would benefit from work examining the links of multiple workplace, healthcare and compensation rehabilitation systems to understand the potential mechanisms for differences in outcomes by injury setting.

Our study observed differences in vocational, disability and functional outcomes, by injury setting in a universal compensation injury compensation scheme, adding little support to the commonly-raised hypothesis that it is differences in entitlement to compensation that explain previously-observed differences in recovery outcomes by injury setting. Many studies have demonstrated a compensation status effect, where entitlement to compensation is related to poor health outcomes [31]. Additionally, compensation scheme legal and administrative processes have been associated with poor outcomes following injury [31]. While it is accepted that the ACC scheme offers administrative efficiencies by managing both work and non-work-related injury claims for all New Zealanders [32] it is possible that scheme administration processes, such as case management, could differ between the two groups. If so, such residual confounding could explain our findings. Worker experiences of the compensation system may also differ by injury setting.

The main strength of this study is the selection of our cohort from a single, universal no-fault injury compensation and rehabilitation provider, allowing for the comparison non-work-related injury group to be selected from the same injury rehabilitation and compensation provider. It is the first time we are aware of that this comparison has been undertaken between two groups with the same entitlement to injury compensation within the same scheme. While this analysis was intended to test a hypothesis by taking advantage of the design of the ACC compensation scheme it is also a limitation as these findings are potentially not generalizable beyond similar no-fault compensation schemes. Further strengths of this study are the inclusion of a broad range of injury types, covering a broad population perspective of the injury burden, giving this study wider generalisability than previous studies. Examination of multiple outcomes prospectively collected across a spectrum of social, vocational, physical and psychological outcome measures, the prospective study design, a large sample size, and adjustment for a range of potential socio-demographic, pre-existing health, injury and compensation factors are also strengths.

Recruitment bias is a possible limitation of this study with the possibility of differential recruitment of workers with work and non-work-related injuries. However, the proportion of workers with work-related injuries recruited into the POIS cohort reflects the proportion of new claimants into ACC with work-related injuries (35% POIS cohort; 32% ACC entitlement claimants [33]) suggesting any recruitment bias by injury setting would be minimal. Further limitations include the use of two similar, but different, measures of work status at the 3- and 12-month time points which introduces the possibility of misclassification of vocational outcomes, although the effect of this difference is likely to be very small. The use of 'subjective’ pre-injury characteristics recalled at the 3 month baseline interview possibly introduced recall bias. Previous analysis of POIS data indicates, however, that estimation of health status prior to injury using retrospective recall of general health status is more appropriate than applying population norms [34]. The unavailability of a comparable pre-injury psychological distress measure using the Kessler-6 tool is a limitation; however, examination of prior depressive episodes using screening questions based on the DSM III reveals no difference in pre-injury psychological status by injury setting. The inclusion of pre-injury disability and functional outcome measures to control for pre-existing disability is a strength for analyses considering these recovery outcomes.

As this study represents the first time the financial gain hypothesis has been tested in the context of universal entitlement these findings should be confirmed in another study using a population with equivalent entitlement to compensation. For example, a number of outcomes examined at both 3- and 12-months following injury had confidence intervals just including the null value, suggesting these outcomes may be important and are thus worthy of future study to confirm if these outcomes differ for workers by injury setting. Additionally, further investigation of the possible time-dependent pattern of recovery by injury setting is warranted with possible consideration of outcomes beyond 12 months, as well as research to understand potential differences in the predictors of outcomes by injury setting. Comparison of outcomes by injury setting, independent of differences in entitlement to compensation, allows for further insight into the workplace, healthcare and compensation rehabilitation system factors associated with work-related injury. Therefore, if confirmed, the policy implications of our findings have utility beyond the New Zealand context. An improved understanding of differences in the outcomes by injury setting would enable workers compensation rehabilitation systems to further understand the importance of policies and practices beyond entitlement to workers compensation in improving recovery outcomes for those workers with work-related injury.

## Conclusion

In this study we hypothesised there would be no difference in outcomes by injury setting for workers within New Zealand’s universal rehabilitation and compensation context as the entitlement for compensation is equivalent regardless of injury setting. Our study found no evidence to reject this hypothesis at 3 months following injury, however by 12 months following injury, the setting in which the injury occurred did influence recovery, with individuals with work-related injuries being more likely to experience limitations impairing work participation and functional ability. To inform future interventions our findings, combined with strong theoretical grounds, indicate that other reasons, beyond differences in compensation entitlement, should be investigated to understand why workers with work-related injury have poorer recovery 12 months following injury.

## Competing interests

The authors declare that they have no competing interests.

## Authors’ contributions

RL prepared the manuscript and is guarantor of this paper. RL and GD analysed the data. All authors contributed to the study design, analysis plan, interpretation of results, and the reviewing and editing of the manuscript. All authors approved the final manuscript.

## Pre-publication history

The pre-publication history for this paper can be accessed here:

http://www.biomedcentral.com/1471-2458/13/995/prepub
